# Can Fetuin A Be Utilized in the Evaluation of Elderly Patients with Acute Myocardial Infarction?

**DOI:** 10.3390/life11090968

**Published:** 2021-09-15

**Authors:** Raluca Tomoaia, Ruxandra Ștefana Beyer, Dumitru Zdrenghea, Alexandra Dădârlat-Pop, Mircea Ioachim Popescu, Gabriel Cismaru, Gabriel Gușetu, Gyorgy Bodisz, Maria Ioana Chețan, Dana Pop

**Affiliations:** 1 Cardiology Department, Heart Institute “N. Stăncioiu”, 400001 Cluj-Napoca, Romania; anda_bogdan@yahoo.com (R.Ș.B.); dadarlat.alexandra@yahoo.ro (A.D.-P.); ioana.chetan@umfcluj.ro (M.I.C.); 2 5th Department of Internal Medicine, Faculty of Medicine, “Iuliu Hațieganu” University of Medicine and Pharmacy, 400012 Cluj-Napoca, Romania; dumitru.zdrenghea@umfcluj.ro (D.Z.); cismaru.gabriel@umfcluj.ro (G.C.); gusetu.gabriel@umfcluj.ro (G.G.); Pop-dana.pop@umfcluj.ro (D.P.); 3 Department of Cardiology, Clinical Rehabilitation Hospital, 400347 Cluj-Napoca, Romania; 4 Department of Medical Disciplines, Faculty of Medicine and Pharmacy, University of Oradea, 410073 Oradea, Romania; procardia_oradea@yahoo.com; 5 Cardiology Department, Clinical County Emergency Hospital of Oradea, 410169 Oradea, Romania; 6 Department of Laboratory Medicine, Clinical Rehabilitation Hospital, 400347 Cluj-Napoca, Romania; gbodizs@yahoo.com

**Keywords:** Fetuin-A, acute myocardial infarction, percutaneous coronary intervention, ST segment resolution, NT-proBNP, troponin

## Abstract

Background: Lower baseline Fetuin-A (FA) is associated with left ventricular remodeling and cardiovascular death (CVD) at 4 months after acute myocardial infarction (AMI). However, the association between FA levels, incomplete ST segment resolution (STR) following primary percutaneous coronary intervention (PCI) and early mortality in AMI has not been previously studied. Methods: We enrolled 100 patients with AMI, which we divided in two groups: 21 patients who suffered sudden cardiac death (SCD) in the first 7 days after PCI and 79 controls. We measured FA, NT-proBNP and troponin levels and correlated them with the occurrence of death in the first week after revascularization. We also tested the cut-off value of FA to determine STR at 90 min after PCI. Results: SCD was most frequently caused by pump failure (n = 10, 47.6%) and ventricular arrhythmias (n = 9, 42.5%). Plasma FA levels correlated with NT-proBNP values (r = −0.47, *p* = 0.04) and were significantly lower in patients presenting SCD (115 (95–175) vs. 180 (105–250) ng/mL, *p* = 0.03). Among all three biomarkers, FA was the only one associated with incomplete STR after PCI on the multivariate logistic regression (cut-off value of 175 ng/mL, Se = 74%, Sp = 61.1%). Death rate was highest (n = 16/55, 30%) in patients with FA levels below the cut-off value of 175 ng/mL. Conclusion: Lower FA is associated with higher early mortality and incomplete STR after primary percutaneous revascularization in patients with AMI. Measurement of FA levels in addition to NT-proBNP, troponin and STR might enable more accurate identification of high-risk patients.

## 1. Introduction

Acute myocardial infarction (AMI) is characterized by myocardial necrosis due to prolonged irreversible ischemia caused by coronary artery occlusion. Researchers have described various mechanisms in the development of AMI, such as rupture of a vulnerable plaque, inflammation or hypercoagulable states, but atherosclerosis remains the major cause [1]. Even though the greater use of reperfusion strategies consisting of primary percutaneous coronary intervention (PCI) and antithrombotic therapy have improved survival in AMI, cardiac arrest (CA) may still occur during the acute phase of AMI. Mechanisms described in the occurrence of sudden cardiac death (SCD) involve myocardial stunning, incomplete reperfusion or an extended area of necrosis. The association of intense antithrombotic treatment with direct stenting of the lesion is imperative in order to avoid distal embolization of the treated vessel [2]. The main causes of CA in the acute phase of AMI are myocardial dysfunction with subsequent heart failure, ventricular arrhythmias and mechanical complication causing cardiogenic shock [3]. Regarding electrocardiographic characteristics, previous studies demonstrated that early ST segment resolution (STR) at 90 min after PCI can predict infarct size, patency of the revascularized vessel, left ventricular ejection fraction (LVEF) and mortality [4].

α2-Hermans-Schmidt glycoprotein or Fetuin-A (FA) is an anti-inflammatory mediator, which is almost exclusively synthetized by hepatocytes [5,6,7]. Previous studies demonstrated that plasma levels of FA decrease during inflammatory processes. Since FA acts by enhancing the inhibition of pro-inflammatory cytokine synthesis, it facilitates the healing process by preventing the self-amplification of inflammatory response [8]. Besides anti-inflammatory properties, FA also has a role in the inhibition of ectopic calcifications by increasing the solubility of calcium and phosphorus in plasma [9]. Moreover, FA might also be involved in insulin resistance [10].

More recently, FA has received attention as a factor linked to cardiovascular mortality. Low levels of FA are associated with a higher severity of CAD [11]. Previous studies revealed significantly lower plasma levels of FA in patients with AMI compared to healthy controls [12]. Regarding patients with STEMI, it was demonstrated that FA levels were significantly more decreased in the first days of admission [13]. Moreover, FA measured 2 days after STEMI reperfused by primary PCI was associated with extent of myocardial necrosis and LVEF after 4 months in cardiac magnetic resonance (CMR) [14]. These findings highlight that myocardial ischemia leads to an inflammatory process [15]. Therefore, increased myocardial calcification and fibrosis associated with low plasma levels of FA might play an important role in the development of LV dysfunction and remodeling.

The aims of this study were:

(1) To measure FA plasma concentrations in patients with AMI after PCI and to test the correlation with other parameters (cardiovascular risk factors, NT-proBNP, troponin);
(2) To test the cut-off value of FA which might predict incomplete STR in patients with STEMI.

## 2. Materials and Methods

### 2.1. Study Population

After approval of the Human Research Ethics Committee and after informed consent of all patients was obtained, 100 patients with AMI, who were admitted to our Cardiology Department from July to September 2019, were enrolled in the study. All patients underwent PCI as the primary strategy of reperfusion. Patients with previously diagnosed coronary artery disease, moderate to severe valvular heart disease, chronic inflammatory diseases, acute systemic infections, liver disease or severe renal failure (eGFR < 30 mL/min/1.73 m^2^) were excluded. On the subjects enrolled, age, medical history and cardiovascular risk factors were recorded.

### 2.2. Biological Measurements

Venous samples were collected immediately upon admission. Plasma was collected in ethylendiamide-tetracetic (EDTA) vials and centrifuged at 4 °C at 3000 rpm for 10 min. For later analysis, vials were stored at −80 °C. Both FA and amino-terminal-pro-brain-natriuretic-peptide (NT-proBNP) plasma levels were measured by sandwich enzyme-linked immunosorbent assay (ELISA): Fetuin-A (Quantikinine ELISA, DFTA00, R&D Systems, Minneapolis, MN, USA), NT-proBNP (ELISA SK-1204, Biomedica Immunoassays, Vienna, Austria). Values were expressed as ng/mL for FA and pg/mL for NT-proBNP. The minimum detection level of FA was 1.74 ng/mL. Other measurements were routine laboratory tests.

### 2.3. Electrocardiographic, Echocardiographic and Angiographic Data

The maximum ST segment elevation and the percent of resolution at 90 min after PCI were recorded in all patients. The cut-off for complete STR was ≥ 70% (3). Echocardiography was performed on a Vivid E95 scanner (GE Vingmed Ultrasound AS, Horten, Norway) using a 2D matrix array transducer (M5S, GE Vingmed Ultrasound AS, Horten, Norway) by observers who were blinded to all clinical and angiographic data. LVEF was calculated using modified Simpson’s biplane formula according to current recommendations. The culprit vessel and the presence of multi-vascular coronary artery disease were recorded on angiography. Coronary blood flow patterns before and after primary PCI were evaluated using the TIMI flow grade.

### 2.4. Statistical Analysis

Statistical analysis was performed using R Core Team 2019 (Vienna, Austria) and Microsoft Excel for Mac 2011. Continuous variables were expressed as mean ± standard deviation (mean ± SD) or median (IQR), according to the distribution of the data. Normality was tested using the Kolmogorov–Smirnov test. Continuous data were compared using *t*-tests or ANOVA tests if the distribution was normal and Mann–Whitney U test or Kruskal–Wallis test otherwise. Categorical tests were compared using chi-square tests. Correlations between variables were assessed using Spearman’s correlation coefficient and the statistical significance was tested with *t*-tests. Multivariate regression analysis was applied for the prediction of incomplete STR after revascularization using the values of FA, NT-proBNP and troponin T. Analysis receiver operating curves (ROC) were performed to assess the area under the curve and cut-off points for FA. A *p* value of <0.05 was considered significant.

## 3. Results

### 3.1. Patient Characterstics

Clinical data of the patients are shown in Table 1. Mean age of the patients was 67 ± 13.1 years and 43% were female. Among cardiovascular risk factors, arterial hypertension (76%), obesity (49%) and smoking (39%) were most frequent. A total of 59% of the patients presented with STEMI on admission. The most frequently involved culprit vessel was LAD (48%), followed by RCA (35%).

Out of all patients, 21% died. The main cause of death was pump failure (n = 10, 47.6%), followed by ventricular arrhythmias (n = 9, 42.5%), while two patients (9.5%) died due to mechanical complications. Patients with AMI presenting CA were older (72 ± 10.8 vs. 65.6 ± 13.4 years, *p* = 0.03) and demonstrated a more reduced LVEF compared to the control group (40 ± 11 vs. 48 ± 10 %, *p* = 0.006), as seen in Table 2. Subjects suffering CA were more frequently diagnosed with STEMI than NSTEMI (19 patients, 90% vs. 50 patients, 63%, *p* = 0.03) and showed higher maximum ST segment elevation on the electrocardiogram before PCI. The majority of the patients had several risk factors, but patients presenting CA did not present significantly more risk factors than controls. A high percent of patients with CA showed incomplete STR or reduced TIMI flow grade after PCI. The majority of the patients had multi-vessel coronary artery disease (>50% stenosis in another coronary artery than the culprit vessel).

### 3.2. Correlation of FA with Other Parameters

Plasma glucose and transaminases were significantly higher in the group presenting CA, but none of these correlated significantly with FA values in this group (Table 3).

Plasma FA levels were significantly lower (115 (95–175) vs. 180 (105–250) ng/mL, *p* = 0.03), while NT-proBNP and troponin levels were significantly higher in patients with AMI presenting CA (1750 (800–3200) vs. 480 (155–945), *p* < 0.001 for NT-proBNP and 0.7 (0.15–2.1) vs. 0.2 (0.07–0.6), *p* = 0.01 for troponin). Plasma FA levels correlated inversely with NT-proBNP levels in the group presenting CA (r = −0.47, *p* = 0.04).

Furthermore, the presence of diabetes mellitus (DM) and the values of triglycerides were correlated to FA levels in patients with CA (r = −0.51, *p* = 0.02 and r = 0.50, *p* = 0.02).

### 3.3. Prognostic Sensitivity and Specificity of FA with Regard to STR at 90 Min after PCI

**Figure 1:**
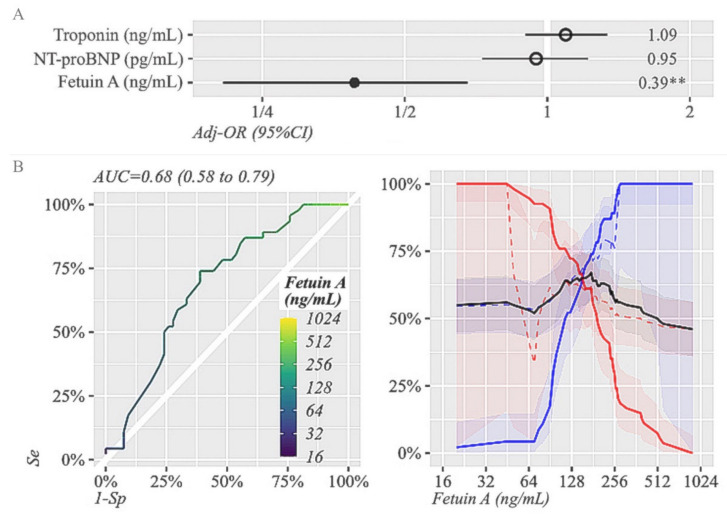
(**A**). Multivariate logistic regression for the adjusted odds ratio of incomplete STR predicted by Fetuin-A, NT-proBNP and Troponin (log2 transformed). *p*-value code ** means *p* < 0.01 (*p*-0.002). (**B**). ROC curve showing the prognostic sensitivity and specificity of Fetuin-A with regard to STR at 24 h after successful PCI. The AUC of Fetuin-A (0.68, 95% CI 0.58–0.79) with the optimal cut-off value of 175 ng/mL, which provided Se = 74% and Sp = 61.1% for incomplete STR. The corresponding NPV and PPV were 73.3% and 62% (black line—plasma levels of the biomarker, Se—blue line, Sp—red line, blue dashed lines–NPV, red dashed lines–PPV). AUC, area under the curve; NPV, negative predictive value; PPV, positive predictive value; OR, odds ratio; ROC, receiver operating curves; Se, sensitivity; Sp, specificity; STR, ST-segment resolution.

We performed a multivariate logistic regression for the adjusted odds ratio of incomplete STR predicted by FA, NT-proBNP and troponin in patients with STEMI, as shown in Figure 1A. Out of all three biomarkers, FA was the only one able to predict STR at 90 min after PCI (*p* = 0.002). The ROC curves showing the prognostic sensitivity and specificity of FA with regard to STR are shown in Figure 1B. The cut-off levels for plasma levels of FA to afford the highest degree of sensitivity (Se) and specificity (Sp) for incomplete STR were 175 ng/mL (Se = 74%, Sp = 61.1%), indicating a moderate accuracy (AUC of 0.68). In contrast to patients with higher levels of FA, patients with lower FA presented more frequently with incomplete STR.

The differences among patients according to the obtained cut-off value of FA are depicted in Table 4. Patients with FA ≤ 175 ng/mL in the acute setting of AMI presented more frequently with CA (16/55 patients-30% vs. 5/45 patients-11%, *p* = 0.03), more frequently had multi-vascular coronary artery disease (36 patients, 65% vs. 19 patients, 42%, *p* = 0.02) and had significantly more elevated NT-proBNP values (910 (320–2250) vs. 420 (130,700), *p* < 0.001) than controls.

## 4. Discussion

Our study demonstrates that low FA levels are associated with higher early mortality and incomplete STR in patients with AMI undergoing primary PCI. The majority of the patients in our study had several risk factors, but patients who died did not present significantly more risk factors. Our findings are consistent with those of Chen et al., who showed that lower plasma FA was associated with increased risk of all-cause mortality and CVD mortality in patients with CAD independently of other cardiovascular risk factors [16], probably due to a high incidence of cardiovascular risk factors among this population. However, we found a moderate correlation between lower FA levels and the presence of DM in patients presenting CA. This association was confirmed by two other studies [10,17], which showed that although FA is an inhibitor of insulin receptor tyrosine kinase that might be involved in insulin resistance, it could also facilitate atherosclerosis beyond the modulation of this mechanism. Although Ix et al. described an association between FA and the lipid profile [11], we only found a relationship between FA and triglycerides in the group of patients with CA, where hypertriglyceridemia was inversely correlated to FA levels. These results were different from those of Weikert et al., who reported elevated FA levels in all patients with modified lipid profiles as a consequence of increased FA liver synthesis induced by the metabolic syndrome [18]. The absence of correlation with cholesterol levels in the whole group might be explained by the fact that some of the patients were undergoing lipid-lowering treatment [19].

Patients with CA were more frequently diagnosed with STEMI and showed higher maximum ST segment elevation on the electrocardiogram on admission. Moreover, a high percent of patients who died showed incomplete STR or reduced TIMI flow grade after PCI and had multi-vessel coronary artery disease, confirming the association between low levels of FA and a higher severity of CAD [11]. Mechanisms described in the occurrence of SCD after AMI involve myocardial stunning, incomplete reperfusion or extended area of necrosis [3] and our study confirmed this data. Even though the greater use of PCI and antithrombotic therapy has improved survival in AMI, CA may still occur during the acute and sub-acute phases [20]. In the recent literature, there has been increasing interest in the role of inflammation in the physiopathology of AMI; thus, several proinflammatory biomarkers have demonstrated their capacity to predict cardiovascular risk and adverse outcomes [21]. Myocardial ischemia enhances the inflammatory process in patients with AMI, as inflammation continues in the remaining myocardium even after completeness of necrosis [15]. During this process, pro-inflammatory cytokines inhibit the synthesis of FA in the liver. Consequently, low FA levels will facilitate the ongoing inflammation and production of cardio-toxic cytokines, such as TNF alpha [22], leading to harmful consequences (LV dilatation, HF, mechanical complications) [23,24,25,26].

We demonstrated that while FA levels were lower, NT-proBNP and troponin levels were significantly higher in acute myocardial infarction. Furthermore, lower FA showed a moderate correlation with elevated NT-proBNP. In the study of Lim et al., low FA levels measured on the third day after STEMI did not correlate to peak cardiac troponin, but did also inversely correlate to NT-proBNP [27]. Furthermore, FA levels were found in the same study to be lower in patients presenting with HF and reduced LVEF, demonstrating that decreased levels of FA play an important role in the pathogensis of HF.

Regarding patients with STEMI, it was demonstrated that FA levels are significantly more decreased in the first days of admission; Cubedo et al. showed that the decline in FA early after STEMI is correlated to the degree of myocardial necrosis [13]. Since it is has been previously demonstrated that early incomplete STR after PCI can predict infarct size, patency of the culprit vessel and mortality [4], we aimed to identify which of the three biomarkers (FA, NT-proBNP and troponin) could predict incomplete STR at 90 min after PCI in patients with STEMI. Lower FA was the only one significantly associated with incomplete STR at 90 min after PCI, even in subjects with preserved or mildly reduced LVEF. Interestingly, in the study of Feistritzer et al., lower FA at 2 days, but not at 4 months after AMI, was associated with infarct size. Thus, this study demonstrated that a single time point FA at baseline might be useful to predict infarct size, indicating the state of inflammatory imbalance and offering information not provided by NT-proBNP or troponin. We found a cut-off level of 175 ng/mL, which showed a moderate accuracy in the detection of STR, and death rate was highest in patients with FA levels below this value. Since both incomplete STR [4] and significantly lower plasma levels of FA measured 2 days after STEMI are correlated with the extent of myocardial necrosis [14], quantification of both these parameters in clinical practice might bring an additional benefit in the identification of patients at risk of unfavorable outcome.

## 5. Limitations

Since the primary limitation of the present study is the limited number of patients, our findings should be confirmed in larger studies. Even though we found correlations between variables irrespective of the value of LVEF, most of the patients had a preserved or only mildly reduced LVEF. This could be a limitation since there were few patients with a severely impaired LVEF. Furthermore, the association between FA and other markers of inflammation might be the subject of a future study. Although incomplete STR at 90 min after PCI can predict infarct size, patency of the revascularized vessel, LVEF and mortality, cardiac magnetic resonance imaging (MRI) remains the gold standard in the evaluation of infarct size and incomplete myocardial reperfusion [28]. Therefore, future studies should focus on finding a cut-off value of FA depending on the extent of necrosis assessed at cardiac MRI.

## 6. Conclusions

In conclusion, lower FA is associated with higher early mortality and incomplete STR after primary percutaneous revascularization in patients with AMI. Measurement of FA levels in addition to NT-proBNP, troponin and STR might enable more accurate identification of high-risk patients.

## Figures and Tables

**Table 1:** General characteristics of the patients.

Variable	Value
Age, years (mean ± SD)	67 ± 13.1
Arterial hypertension (%)	76
Smoking (%)	39
Diabetes (%)	31
Obesity (%)	49
Biological values (mean ± SD or median [IQR])	
Total cholesterol, mg/dL	183.1 ± 51.1
LDL cholesterol, mg/dL	109.5 ± 45.7
HDL cholesterol, mg/dL	42.7 ± 11.5
Triglycerides, mg/dL	155.4 ± 80.4
Glucose, mg/dL	137.1 ± 50
GFR, ml/min/1.73 m^2^	58 ± 19.9
ASAT, mg/dl	36.5 (22.3–70.6)
ALAT, mg/dl	24.5 (17–35.25)
Troponin, ng/mL	0.24 (0.09–0.9)
NT-proBNP, pg/mL	1750 (180–1638)
Fetuin-A, ng/mL	155 (100–241.3)
Type of myocardial infarction	
STEMI (%)	59
NSTEMI (%)	41
Changes in ST segment elevation	
Maximum ST segment elevation, mm (mean ± SD)	2.5 ± 2.5
Complete STR after revascularization (%)	52
Culprit vessel	
LAD, (%)	48
CX, (%)	14
RCA, (%)	35
LM, (%)	3
>1 diseased vessel (n%)	58
TIMI grade flow grade < 3 after revascularization (%)	30
LVEF, % (mean ± SD)	46 ± 10
Causes of death Ventricular arrhythmias, n (%)	9 (42.5)
Mechanical complications, n (%)	2 (9.5)
Pump failure, n (%)	10 (47.6)
Rhythm on cardiac arrest	
Ventricular tachycardia/ ventricular fibrillation, n (%)	12 (57%)
Asistole, n (%)	9 (43%)

ALAT, alanine-aminotransferase; ASAT, aspartate-aminotransferase; CA, cardiac arrest; CX, circumflex coronary artery; eGFR, estimated glomerular filtration rate; HDL, high-density lipoprotein; LAD, left anterior descending coronary artery; LDL, low-density lipoprotein; LM, left main coronary artery; LVEF, left ventricular ejection fraction; NSTEMI, non-ST-elevation myocardial infarction; RCA, right coronary artery; STEMI, ST-elevation myocardial infarction; STR = ST-segment resolution.

**Table 2:** Differences in characteristics between patients with CA and survivors after AMI.

Variable	Patients with CA (n = 21)	Survivors after AMI (n = 79)	*p* Value
Age, years (mean ± SD)	72 ± 10.8	65.6 ± 13.4	0.03 *
Arterial hypertension (n, %)	14 (66.6)	62 (78.5)	0.4
Smoking (n, %)	11 (52.4)	28 (35.4)	0.23
Diabetes (n, %)	4 (19)	27 (34.2)	0.3
Obesity (n, %)	9 (42.8)	40 (50.6)	0.07
Biological values (mean ± SD or median (IQR))			
Total cholesterol, mg/dL	169.2 ± 40.8	186.9 ± 53.2	0.25
LDL cholesterol, mg/dL	100.6 ± 35.4	111.8 ± 48	0.5
HDL cholesterol, mg/dL	40 ± 11.76	43.2 ± 11.4	0.12
Triglycerides, mg/dL	150.8 ± 67.6	156.6 ± 83.77	0.9
Glucose, mg/dL	160.3 ± 63	131 ± 33.2	0.01 *
GFR, mL/min/1.73 m^2^	55 ± 16.6	59.3 ± 18.8	0.45
ASAT, mg/dL	79 (37–130)	28 (21–50)	0.005 *
ALAT, mg/dL	35 (18–67)	23 (17–33.5)	<0.001 *
Troponin, ng/mL	0.7 (0.15–2.1)	0.2 (0.07–0.6)	0.01 *
NT-proBNP, pg/mL	1750 (800–3200)	480 (155–945)	<0.001 *
Fetuin-A, ng/mL	115 (95–175)	180 (105–250)	0.03 *
Electrocardiography			
Type of myocardial infarction			
STEMI, n (%)	19 (90)	50 (63)	0.03 *
NSTEMI, n (%)	2 (10)	29 (37)	
Changes in ST segment elevation			
Maximum ST segment elevation, mm (mean ± SD)	2.5 ± 2.5	1.23 ± 1.4	0.04 *
Complete STR after revascularization (%)	47.6	53	0.13
Coronarography			
Culprit vessel			
LAD, n (%)	11 (52)	37 (47)	0.55
CX, n (%)	1 (5)	13 (6)	
RCA, n (%)	8 (38)	27 (34)	
LM, n (%)	1 (5)	2 (3)	
>1 diseased vessel (n, %)	15 (71)	43 (55)	0.08
TIMI grade flow grade < 3 after revascularization (n, %)	11 (52)	19 (24)	0.03 *
LVEF, % (mean ± SD)	40 ± 11	48 ± 9	0.006 *

ALAT, alanine-aminotransferase; ASAT, aspartate-aminotransferase; CA, cardiac arrest; CX, circumflex coronary artery; eGFR, estimated glomerular filtration rate; HDL, high-density lipoprotein; LAD, left anterior descending coronary artery; LDL, low-density lipoprotein; LM, left main coronary artery; LVEF, left ventricular ejection fraction; NSTEMI, non-ST-elevation myocardial infarction; RCA, right coronary artery; STEMI, ST-elevation myocardial infarction; STR = ST-segment resolution. * Statistically significant.

**Table 3:** Correlations between Fetuin A levels and other parameters.

Variable	All Patients	Patients with CA	Survivors after AMI
Age, years	−0.21 (*p* = 0.02)	NS	NS
Arterial hypertension, %	NS	NS	NS
Smoking, %	NS	NS	NS
Diabetes, %	NS	−0.51 (*p* = 0.02)	NS
BMI, kg/m^2^	NS	NS	NS
Total cholesterol, mg/dL	NS	NS	NS
LDL cholesterol, mg/dL	NS	NS	NS
HDL cholesterol, mg/dL	NS	NS	NS
Triglycerides, mg/dL	0.22 (*p* = 0.02)	0.50 (*p* = 0.02)	NS
Glucose, mg/dL	0.28 (*p* = 0.004)	NS	0.40 (*p* < 0.01)
eGFR, mL/min/1.73 m^2^	NS	NS	NS
ASAT, mg/dL	NS	NS	NS
ALAT, mg/dL	NS	NS	NS
Troponin, ng/mL	NS	NS	NS
NT-proBNP, pg/mL	−0.31 (*p* = 0.001)	−0.47 (*p* = 0.04)	NS
LVEF, %	NS	NS	NS

ALAT, alanine-aminotransferase; ASAT, aspartate-aminotransferase; BMI, body mass index; CA, cardiac arrest; eGFR, estimated glomerular filtration rate; HDL, high-density lipoprotein; LDL, low-density lipoprotein; LVEF, left ventricular ejection fraction.

**Table 4:** Comparison between groups according to cut-off value of Fetuin-A.

Variable	FA ≤ 175 ng/mL (n = 55)	FA > 175 ng/mL (n = 45)	*p*
Age, years	68 ± 14	66 ± 12	0.40
Male sex, n (%)	33 (60%)	24 (53%)	0.50
NT-proBNP, pg/ mL	910 (320,2250)	420 (130,700)	<0.001 *
Troponin, ng/ mL	0.3 (0.13–1.1)	0.22 (0.07–0.8)	0.18
LVEF, %	44 ± 11	48 ± 8	0.07
Number of diseased vessels > 1, n (%)	36 (65)	19 (42)	0.02 *
TIMI flow grade < 3, n (%)	20 (36%)	10 (22%)	0.12
Number of patients with CA, n (%)	16 (30)	5 (11)	0.03 *

CA, cardiac arrest; LVEF, left ventricular ejection fraction; STR, ST-segment resolution. * Statistically significant.

## Data Availability

The data that support the findings of this study are available from the corresponding author upon request.
